# ACTIV trials: cross-trial lessons learned for master protocol implementation

**DOI:** 10.1017/cts.2024.507

**Published:** 2024-10-15

**Authors:** Maryam Keshtkar-Jahromi, Stacey J. Adam, Indira Brar, Lucy K. Chung, Judith S. Currier, Eric S. Daar, Victoria J. Davey, Eileen T. Denning, Annetine C. Gelijns, Elizabeth S. Higgs, Prasanna Jagannathan, Arzhang Cyrus Javan, Tomas O. Jensen, Nikolaus Jilg, Ioannis Kalomenidis, Peter Kim, Seema U. Nayak, Matthew Newell, Babafemi O. Taiwo, Tammy Yokum, Yvette Delph

**Affiliations:** 1 Division of Microbiology and Infectious Diseases, National Institute of Allergy and Infectious Diseases, National Institutes of Health, Rockville, MD, USA; 2 Foundation for the National Institutes of Health, North Bethesda, MD, USA; 3 Henry Ford Health, Detroit, MI, USA; 4 CAMRIS International (under Contract No. 75N93019D00025) with National Institute of Allergy and Infectious Diseases, NIH, DHHS, Bethesd, MD, USA; 5 JSC Department of Medicine, David Geffen School of Medicine, University of California, Los Angeles, CA, USA; 6 Division of HIV Medicine, Lundquist Institute at Harbor- University of California Los Angeles Medical Center, Torrance, CA, USA; 7 Office of Research and Development, US Department of Veterans Affairs, Washington, DC, USA; 8 Division of Biostatistics & Health Data Science, University of Minnesota School of Public Health, Minneapolis, MN, USA; 9 Population Health Science and Policy, Mount Sinai, New York, NY, USA; 10 Division of Clinical Research, National Institute of Allergy and Infectious Diseases, Department of Health and Human Services, National Institutes of Health, Rockville, MD, USA; 11 Stanford University, Stanford, CA, USA; 12 National Institute of Allergy and Infectious Diseases, National Institutes of Health, Rockville, MD, USA; 13 Centre of Excellence for Health, Immunity, and Infections, Rigshospitalet, University of Copenhagen, Kobenhavn, Denmark; 14 Division of Infectious Diseases, Massachusetts General Hospital, Brigham and Women’s Hospital, Harvard Medical School, Boston, MA, USA; 15 1st Department of Critical Care and Pulmonary Medicine, National and Kapodistrian University of Athens School of Medicine, Evangelismos Hospital, Athens, Greece; 16 Global Health & Infectious Diseases, School of Medicine, University of North Carolina, Chapel Hill, CA, USA; 17 Northwestern University, Evanston, IL, USA; 18 Axle Informatics, North Bethesda, MD, USA

**Keywords:** Accelerating COVID-19 Treatment Interventions and Vaccines, COVID-19, clinical trial implementation, master protocol, preparedness, trial platform

## Abstract

The United States Government (USG) public-private partnership “Accelerating COVID-19 Treatment Interventions and Vaccines” (ACTIV) was launched to identify safe, effective therapeutics to treat patients with Coronavirus Disease 2019 (COVID-19) and prevent hospitalization, progression of disease, and death. Eleven original master protocols were developed by ACTIV, and thirty-seven therapeutic agents entered evaluation for treatment benefit. Challenges encountered during trial implementation led to innovations enabling initiation and enrollment of over 26,000 participants in the trials. While only two ACTIV trials continue to enroll, the recommendations here reflect information from all the trials as of May 2023. We review clinical trial implementation challenges and corresponding lessons learned to inform future therapeutic clinical trials implemented in response to a public health emergency and the conduct of complex clinical trials during “peacetime,” as well.

## Introduction

**Figure 1.**
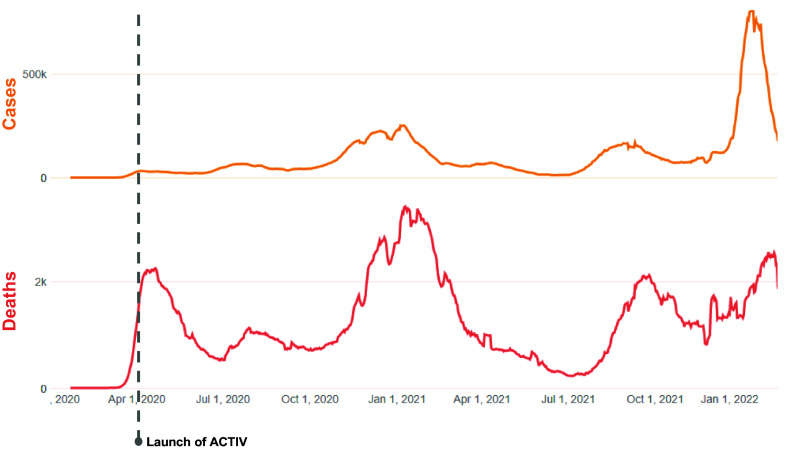
COVID-19 cases and deaths in the US (2020–2022). Permissions received to use the data from Johns Hopkins University Coronavirus Resource Center. https://coronavirus.jhu.edu/region/united-states. ACTIV = Accelerating COVID-19 Treatment Interventions and Vaccines.

On April 17^th^, 2020, when the National Institutes of Health (NIH) launched the United States Government (USG) public-private partnership, Accelerating COVID-19 Treatment Interventions and Vaccines (ACTIV) facilitated by the Foundation for the National Institutes of Health, the US was experiencing its first COVID-19 wave with 2,227 deaths per day (7-day average) from an estimated 28,677 new cases per day (Fig. 1). Hospital intensive care units (ICUs), morgues, and public health infrastructure were not keeping pace with the large number of ill patients and those succumbing to COVID-19. Anxiety and fear were high, particularly in hard-hit cities. Pharmaceutical companies and research institutions across the globe launched clinical trials and cohort studies.

**Figure 2.**
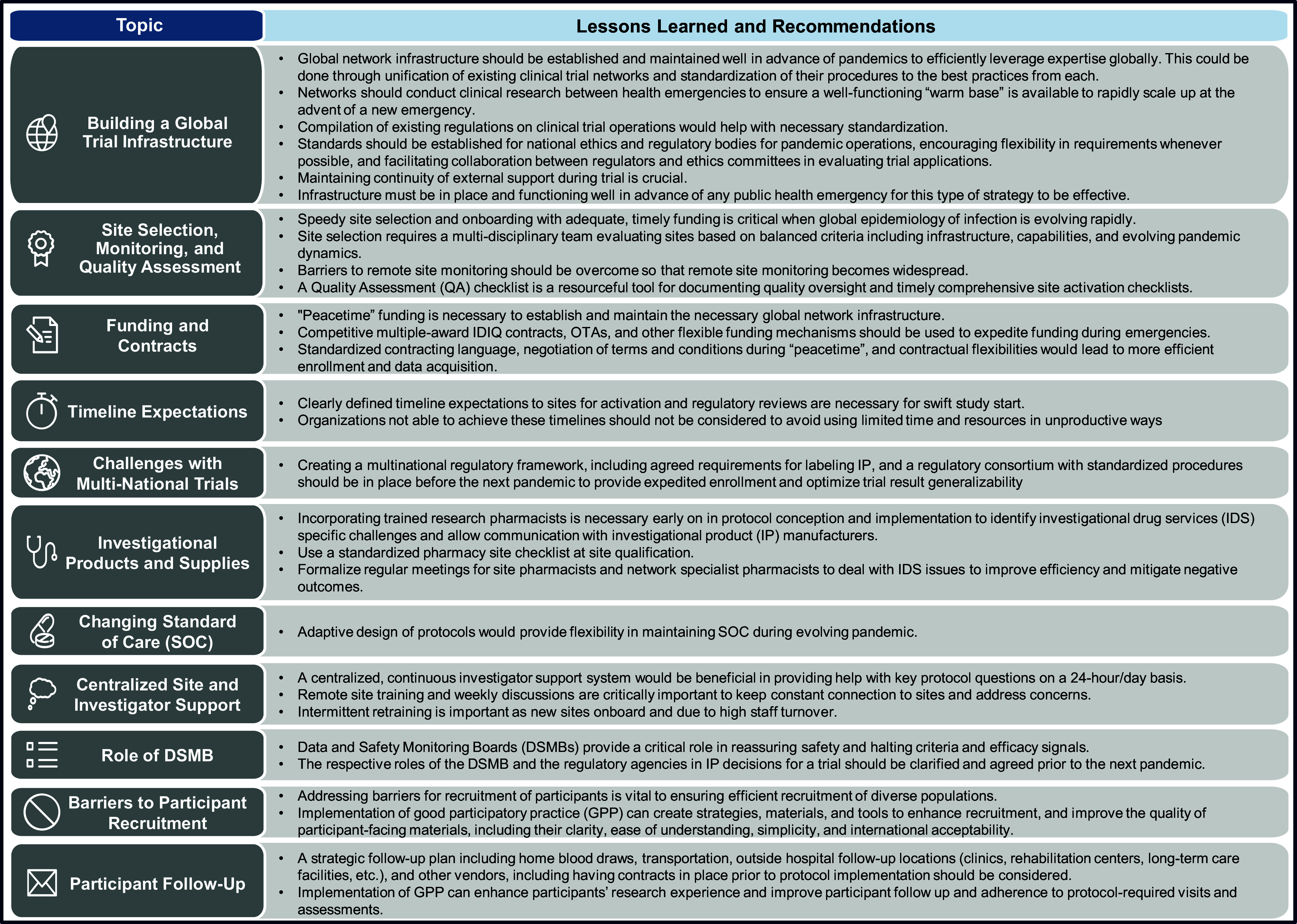
ACTIV master protocol implementation lessons learned: the high-level takeaway lessons learned from the trial implementation of the ACTIV master protocols and potential solutions that may be utilized in future pandemics. ACTIV = Accelerating COVID-19 Treatment Interventions and Vaccines; IDIQ = indefinite delivery indefinite quantity; OTA = other transactional authority; QA = quality assessment; TMF = Trial Master File; IDS = Investigational Drug Services; IP = investigational products; SOC = standard of care; DSMB = Data and Safety Monitoring Board; GPP = good participatory practices.

In this urgent context, the ACTIV Therapeutics-Clinical Working Group (ACTIV TX-Clin WG) began identifying therapeutic candidates and research questions suitable for testing under a master protocol [1]. As defined by the NIH, a master protocol is an approach that tests multiple interventions and/or multiple subpopulations in parallel under a single protocol, without the need to develop new protocols for every trial [2]. Ultimately, the ACTIV TX-Clin WG conceptualized a suite of master protocols based on study population, therapeutic target, and novel versus repurposed agents [3]. To design and implement the master protocols, the ACTIV TX-Clin WG recruited a variety of experienced clinical trial networks to lead and conduct the trials. Selection was based on network experience, scientific ability, operational qualifications, and a track record of designing and implementing regulatory enabling studies. Once initial network leadership accepted responsibility for one of the master protocols, protocols and sub-studies were developed, submitted to the regulatory authorities, and implemented [3]. There were a myriad of implementation challenges, some attributable to the relatively late start of ACTIV studies in comparison to individual organization efforts and absence of available research capacity, others due to overwhelmed hospitals and staff and simple supply shortages due to pandemic supply chain challenges. Ultimately, ACTIV advanced eleven initial master protocols, enrolled more than 26,000 participants, and importantly, evaluated treatment benefits for 37 therapeutic agents. Here we identify key implementation lessons applicable to future preparedness and response efforts (Fig. 2).

## Goals and objectives of ACTIV

With thousands of US deaths daily, the mandate for the ACTIV TX-Clin WG was to design and implement protocols to identify safe therapeutics to treat patients to prevent hospitalization, progression of disease, and death. Each ACTIV protocol team had slightly different goals within this mandate to save lives (Table 1). ACTIV-1 focused on host-targeted therapeutics in hospitalized patients. ACTIV-2 trialed monoclonal antibodies and antiviral agents for outpatients. ACTIV-3 and -3B focused on monoclonal antibodies, antivirals, and immune modulators in hospitalized patients. ACTIV-4A, -4B, and -4C focused on testing anticoagulation therapies and ACTIV-4HT the renin-angiotensin system. ACTIV-5 (also known as the Big Effect Trial) was looking for a large mortality benefit for products that could be fed into ACTIV-1, -3, or -4HT. ACTIV-6 was a decentralized trial with the potential for fully remote testing of repurposed agents started to identify repurposed agents that might have benefit in mild/moderate acute COVID and which might be rapidly implemented (if beneficial) or de-implemented (if not beneficial or if harmful) based on trial results. This allowed ACTIV-6 to address repurposed agents with both public support and scientific equipoise.

**Table 1.** Overview of the ACTIV studies

Population	Master Protocol	Phase	Drug Class	Networks	Total Enrollment and Number of Sites that Enrolled Participants	Participating Countries
**Inpatient Studies**	**ACTIV-1** NCT04593940	III	Host-targeted Immune Modulators	BARDA/NCATS + CTSA Program/TIN + CRO (TRI/DCRI/Syneos)	1,971 total participants 85 sites	Argentina, Brazil, Mexico, Peru, United States
	**ACTIV-3** NCT04843761	III	mAbs and Antivirals	NIAID INSIGHT + NHLBI PETAL + CTSN + VA	2,752 total participants 115 sites	Argentina*, Denmark, Greece, India*, Mexico*, Mozambique*, Nigeria, Poland, Singapore, Spain, Switzerland, Uganda, United Kingdom, United States
	**ACTIV-3B** NCT05780463	III	Host-targeted Immune Modulators and Antivirals for ARDS	NIAID INSIGHT + NHLBI PETAL + CTSN + VA	473 total participants 28 sites	Brazil*, United States
	**ACTIV-4A** NCT04505774	III	Host-tissue Directed Antithrombotics Vascular Integrity/Thromboinflammation	NHLBI CONNECTS	3,184 total participants 122 sites	Brazil, Italy, Mexico, Spain, United States
	**ACTIV-4HT** NCT04924660	II/III	Host-tissue Targeted Therapies	NHLBI CONNECTS	899 total participants 65 sites	Argentina, Brazil, Germany, Italy, South Africa, Spain, United States
	**ACTIV-5** NCT04583969	II	Screen Promising Immune Modulators	NIAID + CRO	821 total participants 57 sites	South Korea, United States
	**STRIVE (severe ARI)** NCT03085888	III	All agents relevant to acute viral respiratory diseases	NIAID INSIGHT	411 total participants (as of May 8, 2024) 159 sites	Argentina, Australia, Belgium, Brazil, Burkina Faso, Cambodia, Cameroon, Canada, Croatia, Denmark, France, Germany Georgia, Greece, Guinea, India, Ireland, Italy, Ivory Coast, Japan, Madagascar, Mexico, Mozambique, Niger, Nigeria, Peru, Poland, Senegal, Singapore, South Africa, South Korea, Spain, Switerzland, Togo, Uganda, Ukraine, United Kingdom, United States, Vietnam
**Outpatient Studies**	**ACTIV-2** NCT04518410	II/III	mAbs, Oral Antivirals, Polyclonal Ab, and Inhaled Interferon	NIAID ACTG + CRO	4,044 total participants 172 sites	Argentina, Brazil, Guatemala, Mexico, Philippines, South Africa, United States
	**ACTIV-2D** NCT05305547	III	Oral Antiviral	NIAID ACTG Shionogi (Study Sponsor) CRO	2,093 total participants 207 sites	Argentina, Brazil, Columbia, Ghana, India, Japan, Kenya, Malawi, Mexico, Pakistan, Philippines, Poland, South Africa, Thailand, Uganda, United States
	**ACTIV-4B** NCT04498273	III	Host-tissue Directed Antithrombotics	NHLBI CONNECTS	657 total participants 50 sites	United States
	**ACTIV-6** NCT05894577	III	Existing Prescription and OTC Medications	NCATS CTSA and Trial Innovation Network + PCORnet + Conduct Clinical Trials + SignalPath	10,767 total participants (as of April 5, 2024) 110 sites	United States
	**ACTIV-4C (convalescent)** NCT04650087	III	Host-tissue Directed Antithrombotics	NHLBI CONNECTS Network	1,217 total participants 106 sites	Argentina, Guatemala, Mexico, Philippines, United States

ACTG = AIDS Clinical Trials Group; ACTIV = Accelerating COVID-19 Therapeutic Interventions and Vaccines; ARDS = acute respiratory distress syndrome; ARI = acute respiratory infection; AZ = AstraZeneca; BMS = Bristol Myers Squibb; CONNECTS = Collaborating Network of Networks for Evaluating COVID-19 and Therapeutic Strategies (42 participating networks and cohorts can be found here: https://nhlbi-connects.org/organizations); CTSN = Cardiothoracic Surgical Trials Network; CRO = contract research organization ; DCRI = Duke Clinical Research Institute; INSIGHT = International Network for Strategic Initiatives in Global HIV Trials ; Lilly = Eli Lilly and Company; mAbs = Monoclonal Antibodies; NCATS = National Center for Advancing Translational Sciences at NIH ; NHLBI = National Heart, Lung and Blood Institute at NIH; NIAID = National Institute of Allergy and Infectious Diseases at NIH; OTC = over-the-counter; PETAL = Prevention and Early Treatment of Acute Lung Injury; PCORnet = National Patient-Centered Clinical Research Network; SGLT2 = Sodium-glucose cotransporter-2; STRIVE = Strategies and Treatments for Respiratory and Viral Diseases; TIN = Trial Innovation Network; TRI = Technical Resources International; Inc; VA = US Department of Veterans Affairs Research Network.

* Countries that had sites registered, but did not randomize any participants.

## Building a coherent global trial infrastructure during the pandemic involved leveraging existing clinical trial capacity

The ACTIV TX-Clin WG interviewed multiple existing NIH-funded clinical trial groups and research sites to determine willingness to collaborate on large trials during the pandemic. Among those willing, the ACTIV TX-Clin WG organized these consortia and designated a “lead” group. [Further information can be found in the Master Protocol Design Lessons Learned Manuscript in this issue.]

For ACTIV-1, the ACTIV TX-Clin WG leveraged the Clinical and Translational Science Awards (CTSAs) Program clinical network overseen by US NIH National Center for Advancing Translational Sciences (NCATS) [4]. The CTSA Program is a standing national network of large medical research institutions – called hubs – that work together through various consortium-wide projects and initiatives such as the Trial Innovation Network (TIN) to improve the overall translational research process to expedite the delivery of treatments to patients. The hubs collaborate locally and regionally to catalyze innovation in training, research tools, and processes. Funding was provided via a subcontract to the Duke Clinical Research Institute (DCRI) from Technical Resources International, Inc. (TRI), the contract research organization (CRO) contracted by the US Biomedical Advanced Research and Development Authority (BARDA). The CTSA program provided the initial US enrollment sites, but it was necessary to rapidly add more community hospitals and other US sites recommended by CTSA Principal Investigators (PIs), NIH, and pharmaceutical partners. A TRI subcontract to Syneos, a CRO, added global sites in Latin America. Ultimately, ACTIV-1 tested three immune modulators (abatacept, cenicriviroc, and infliximab) and enrolled 1,971 participants (1,291 from US sites, 680 from Latin America sites). ACTIV-1 provided a good lesson that pre-established network connections allow for faster trial startup: since its full network was not established, ACTIV-1 took 4 months longer to start than ACTIV-2 and-3. It also provided a model for an academic team to hold the Investigational New Drug (IND) on behalf of the sponsor for the whole trial.

ACTIV-2 leveraged the AIDS Clinical Trials Group (ACTG) infrastructure, with its considerable clinical trialist experience that helped address the HIV pandemic [5], as its base network. The ACTG, founded in 1987 through an NIAID/Division of acquired immunodeficiency syndrome (AIDS) (DAIDS)-funded cooperative agreement, is the world’s largest and longest-running global clinical trials network focused on HIV, which also does research on tuberculosis and viral hepatitis. To implement ACTIV-2, the ACTG engaged a CRO that evaluated and enlisted additional sites, some of which were part of the NIH’s clinical research site infrastructure from other networks overseen by other NIH Institutes. ACTIV-2 used a hybrid model, incorporating some of the ACTG’s usual processes while transferring an extensive number of regulatory obligations and ACTG core responsibilities to the CRO, who played the primary trial implementation role. Data management and central laboratory processes were managed by the CRO, while both CRO and ACTG statisticians oversaw different statistical aspects of the trial. This hybrid design had strengths and weaknesses. Partnering with a large CRO to rapidly onboard clinical trial sites internationally, and the NIH’s network of sites at large, was considered essential to helping the ACTIV-2 platform meet its ambitious enrollment goals. Ultimately,173 sites enrolled 4,043 participants across 7 countries on 4 continents [6]. The study also benefited tremendously from the ACTG network’s scientific leadership and expertise in designing and writing the protocol working closely with the Sponsor, DAIDS, to help oversee the scientific conduct of the trial. Though overall successful, the ACTIV-2 team found that expeditiously building and maintaining new processes that merged the ACTG’s and CRO’s efforts, while needing to rely on the CRO for trial implementation, led to operational complexities that delayed the trial’s ability to receive deliverables and disseminate findings in an optimal timeframe.

ACTIV-3 combined four academic USG-funded trial networks into one consortium, with INSIGHT designated as lead group. The Division of Clinical Research (DCR) at the NIAID [7] also recruited clinical sites to participate in ACTIV-3 and was effectively an additional network. INSIGHT was originally established in the 1990s by building relationships between five experienced academic trial coordinating centers around the world, creating a multinational infrastructure into which additional partners could be integrated quickly when the pandemic emerged. The National Heart, Lung, and Blood Institute (NHLBI) Prevention and Early Treatment of Acute Lung Injury (PETAL) and Cardiothoracic Surgical Trials Network (CTSN) networks, the Veterans Affairs Research Network (VA), and DCR network functioned as additional trial coordinating centers in the INSIGHT distributed coordination model, and representatives of these networks joined the INSIGHT leadership team. The five groups worked as one team beginning in June 2020 by using a common platform protocol and integrating all networks into INSIGHT’s existing data collection system. Ultimately 139 sites in 15 countries registered for at least one ACTIV-3 or 3B trial, and 122 sites in 10 countries in Africa, Asia, Europe, and North America enrolled 3,225 participants. ACTIV-3 provided great lessons for how to merge networks through unifying contracting under one coordinating center and standardizing operational procedures across all networks.

ACTIV-4A established a US-based network through the NHLBI Collaborating Network of Networks for Evaluating COVID-19 and Therapeutic Strategies (CONNECTS) program [8]. It was able to leverage a parallel master protocol platform conducted in an existing international network, Randomized Embedded Multifactorial Adaptive Platform for Community-Acquired Pneumonia (REMAP-CAP) [9], by joining forces with the Antithrombotic Therapy to Ameliorate Complications of COVID-19 trial [10] to form the multi-platform Randomized Clinical Trials (mpRCT). REMAP-CAP was conceived in 2011 as an adaptive platform for trials to treat community-acquired pneumonia. This platform included a “Pandemic Appendix” which was activated early in 2020. The mpRCT collaboration was established just a few months into the establishment of ACTIV-4A and the CONNECTS network for the pandemic. It permitted harmonization of the platform protocols being carried out by each group, including common primary outcomes, similar secondary outcomes, and overlap and similarity in data collection. This global collaboration led to 393 sites in over 10 countries generating results within months and allowing for dramatically enhanced enrollment. ACTIV-4B, -4C, and -4HT also operated within the CONNECTS network but did not pair with other outside trial networks. While ACTIV-3 and ACTIV-4A used different approaches (combining networks to conduct a common platform protocol in a single data collection system vs. harmonizing trial protocols and data collection between networks in independent systems), the ability of existing networks to partner with a preexisting multinational trial structure was critical to both studies. A key to their success is a distributed coordination model that leverages the diverse experiences of academic consortia and skilled research sites across the globe. While there must be a single “lead” center for such an infrastructure, the relationships established among the centers, and of the centers with the sites they oversee, are what provide the capacity to function as a truly global trial network. A twelfth master protocol, Strategies & Treatments for Respiratory infections and Viral Emergencies (STRIVE) launched on February 12, 2023, has begun applying some of the lessons learned in the original ACTIV master protocols and will undertake preparations for the next public health emergency [11,12]. STRIVE has built on the success of ACTIV-3,-3B, -1, and -5 by combining the collaborating networks (CTSN, INSIGHT, PETAL, and VA) into one STRIVE network.

ACTIV-5 used a hybrid model to create a network for the trials conducted within its adaptive protocol, utilizing some sites from existing networks that were still able to engage with new trials (Infectious Disease Clinical Research Consortium, HIV Prevention Trial Network, HIV Vaccines Trial Network, and the network created for the Adaptive Covid Treatment Trial (ACTT)) along with sites evaluated by the study’s CRO. The CRO used case rates from surveillance data to identify potential sites in highly affected areas of the United States. Some sites had less experience with conducting clinical trials, which led to increased training requirements. Some sites required simple updates on working with NIAID as a Sponsor (e.g. roles and responsibilities of our operational offices), while others needed more extensive training on more rudimentary clinical trial practices, such as source documentation standards, adverse event reporting, and use of a single IRB. Creating a new network led to a lack of cohesiveness in trial implementation, despite multiple training modalities, consistent team calls, and dedicated follow-up from CRO and sponsor representatives.

The ACTIV-6 platform benefited from the leadership of the US National Patient-Centered Clinical Research Network (PCORnet) and the CTSA TIN in establishing its initial enrollment footprint. The Conduct Clinical Trials and SignalPath site networks also contributed sites to augment enrollment under the decentralized model. Engagement with new networks and sites in a decentralized trial structure identified unexpected practices and behaviors that required assessment for impact on the research, such as the heavy use of Facebook and other social media platforms for raising awareness of the trial and often serving as the first source of information for interested participants. These new practices did not seem to affect the integrity of the trial conduct but could have led to some bias in subjects enrolled. ACTIV-6 recruited only in the US and engagement with existing networks allowed recruitment of a diverse cohort of participants that included participants from every state in the US. ACTIV-6 provided a lesson for a model that can be replicated for hybrid or fully remote trials during future pandemics.

## Site selection, feasibility assessment

The critical issue for available site capacity is to start a centrally coordinated research response immediately. Many experienced clinical research sites within existing clinical research networks were already conducting pharmaceutical company-driven trials. Clinical research infrastructure needs to have support and policy provisions to pivot from usual research and trial conduct to focus on country-level health emergency priorities for optimal trial efficiency. Sites needed for the ACTIV trials reflected the networks recruited to undertake those studies, e.g. inpatients, outpatients, ICU, etc. The network geographic distribution was critical to all ACTIV trial networks to ensure enrollment of diverse populations and ability to follow the pandemic waves. Multiple groups with diverse backgrounds were needed to prospectively balance needs of following pandemic spread by location and selecting high-performing sites capable of conducting complex clinical trials. Assessing site capabilities primarily focused on past research experience with related trials rather than regional case number, although selection subsequently incorporated regional prevalence of infection. A “network of networks” afforded faster startup using existing collaborations and communications to rapidly build the platform team.

Initial ACTIV studies of non-hospitalized individuals with COVID-19 started selection with ACTG sites and other DAIDS clinical sites allowing use of an established multinational research network experienced in treating non-hospitalized patients. For this group, site selection focused on experience with outpatient studies as well as willingness, commitment, and capability to study a highly infectious pathogen, which required specific facilities and staff. With this foundation, other interested and experienced sites globally were identified using a CRO and other tools [see Master Protocol Design Lesson Learned report.] Sites were responsible for working with protocol leadership to establish an outreach plan and enhance enrollment by utilizing remote and/or touchless procedures. This was subsequently extended to establishing studies of repurposed agents that were fully remote for conduct of ACTIV-6.

## Site quality assurance and monitoring

Sponsor responsibility for quality assurance (QA) and oversight [13] posed challenges when working with sites and CROs new to the networks. ACTIV-5 created a QA checklist to address QA oversight for both sites and sponsors. The checklist required PI attestation to compliance with QA requirements while listing critical protocol implementation elements (consenting, eligibility, adverse event assessment, safety reporting, and deviations). Sites’ value of the QA checklist tool varied depending on whether they had preexisting QA programs. Identifying deviations such as uncompleted protocol procedures were quickly corrected to ensure participants’ safety and data quality. Weekly emails sent to sites noting lessons learned or frequently asked questions helped communicate challenges and solutions.

For some of the ACTIV trials, monitoring teams were tasked with ensuring QA documentation and protocol implementation success was dependent on real-time active monitoring of sites with an emphasis on early monitoring (first 3 participants). One of the major monitoring challenges was doing on-site visits. During the pandemic, many facilities prevented on-site monitoring because of infection control policies that restricted physical access to the site, and it was difficult to find monitors willing and able to travel to sites. As a response, remote monitoring was instituted. This was a time-consuming process for both monitors and site staff, and it brought its own challenges including difficulties with getting access to and reviewing source documents, delays at the site in getting systems and permissions set up timely for monitoring, and concerns the remote monitors were not seeing all the source documents. Some hospital systems did not allow remote access, so “over-the-shoulder reviews” were done with site staff sharing their screens displaying source documents. However, even with these challenges, remote monitoring did allow monitors to broadly cover sites without travel and risk of exposure to the pandemic agent. ACTIV trial teams would recommend remote monitoring, where possible, both in peacetime and the next public health emergency.

## Contract logistics, agreement, and execution

Government contracting processes were lengthy and formidable even in the public health emergency, causing delays in trial implementation. Contractors were concerned about failures to meet federal compliance. NIH support letters to institutional leadership in advance were helpful; however, a more formal requirement for sites to accept terms as is or with minimal negotiation, and standard clauses that contractors are compliant with federal regulations, would streamline contracting. A dedicated investigation into standardized contracting language is warranted for future pandemic deployment. Ability to harness Other Transaction Authorities (OTAs), a flexible US federal contracting mechanism, or pre-negotiated, existing contract mechanisms, such as Indefinite Delivery, Indefinite Quantity (IDIQs) awards, more broadly would be useful. Master subcontracts with task orders by USG contractors would also be time-saving. The prime contractor to the USG could issue a master subcontract with all the terms and conditions already negotiated and agreed upon during peacetime. Task orders could then be issued with just statements of work and funding. Of note, existing networks that were able to harness ongoing contractual relationships with their sites (such as ACTG sites for ACTIV-2 and INSIGHT sites for ACTIV-3) were able to implement studies quickly.

When selecting a full-service CRO, a complete understanding of personnel depth, coverage, flexibility, and recovery plan is crucial to account for attrition across staffing roles. Major CROs were stretched thin and subsequently had fewer experienced individuals. Academic centers also faced similar challenges with limited staff availability, though they had less detailed and rigid procedures and were more adaptable and creative. Contractual flexibility to switch CROs when sponsor needs are not met (e.g., with regards to qualified personnel, timelines, and data systems) could expedite trial conduct along with consequences for poor performance and milestone delinquencies, even though it could cause other types of delays to transition midstream. Additionally, nimble subcontracting is critical for any CRO; timelines for subcontracts between CROs and sites were a major source of delay. An understanding of the CROs’ track record and timelines for executing subcontracts with sites should be a selection criterion. Definition of past subcontracting benchmarks upfront when engaging a CRO could be an important metric for CRO selection in future pandemics. It would be helpful for the USG and other funders/sponsors to pre-qualify CROs using a competitive mechanism such as a multiple-award IDIQ contract, under which task orders could be issued when the need arises.

## Timelines for site activation

While individual research teams may mobilize quickly in a pandemic, institutions adapt more slowly. Because institutional requirements varied substantially between sites, timelines to initiation of enrollment were unclear. Institutions were prioritizing COVID-19 research but struggled to accommodate all desired research.

In the US, even though sites were required to use a single institutional review board (sIRB) [14], many sites had additional local review requirements. This included local IRB reviews, research and development reviews, resource assessments, competing studies evaluations, and marketing and communication approvals for participant-facing materials. Sites also needed their institutions to establish a reliance agreement with the sIRB if one not already executed. The US Department ofHealth and Human Services (HHS) exception to the sIRB requirement, enacted in October 2020, came well after the planning stages of the ACTIV trials, when processes for sIRB adherence had already been put in place [15].

US local IRBs needed education on the sIRB process and ramifications of ceding review to a sIRB, as sIRB reliance is a recent phenomenon. Review by a sIRB at the national level has long been common practice in other countries, but in some, elaborate local IRB requirements still hamper the rapid startup that sIRB review facilitates.

Clear timeline expectations should be established by the funder and sponsor and included in site feasibility assessments, contracts, task orders, and agreements. All parties must be accountable for timelines, and corrective action taken early if they are not met. In a pandemic, organizational ability to expedite contracts, establish site agreements, and accelerate or waive institutional review is paramount.

Site activations posed challenges with required regulatory document collection. A centralized tracking platform (e.g., Smartsheet) improved team members’ review and provided real-time site activation status. Regulatory requirements were streamlined by collecting personnel documents only for PIs for activation; monitors then ensured other study personnel regulatory documents were on-site. Leveraging existing sites within active networks avoided these challenges.

## Challenges with multinational trials

A major challenge was timely regulatory approvals across multiple countries. A multinational regulatory framework would provide expedited enrollment and optimize trial result generalizability. The ACTIV trials started with US Food and Drug Administration (FDA) approvals and then protocols were laboriously submitted country-by-country to other regulatory agencies. Though most regulatory agencies were prioritizing COVID-19 trials, the recruitment period of several trials was brief and many countries could not participate in enrollment because of the required sequential approval approach. In some countries, regulatory processes only prioritized their country’s COVID-19 trials and deprioritized multilateral trials. This problem was amplified because non-US filings only occurred after FDA approval. In some instances, pharmaceutical partners did not have required documents or could not share confidential documents with the network for regulatory submissions. Procedural and capacity variances at regulators worldwide resulted in significant differences in application time and approval. During a pandemic, this is a major impediment to international engagement on a common protocol.

Labeling of study agents to be distributed in multiple countries was also a regulatory challenge. Because a universally regulatorily acceptable investigational product (IP) label does not exist, decisions were made to balance efficient and adaptable distribution with multiple country regulatory requirements. Country-specific language requirements were a common logistical barrier to study agent label development, and several options were considered to address country-specific needs. For example, labeling entire study stock with a multi-panel booklet containing compliant labeling for each country maintains flexibility for distribution, but substantially increases label development time before study drug can be shipped to a central distributor. If instead, multiple drug stocks are maintained and labeled with country-specific content, flexibility to modify distribution plans and re-allocate drug is limited. Given timeline variability for site readiness between countries and limited agent supply, having multiple mutually exclusive stocks could have resulted in delays or inefficient distribution. Other options such as applying auxiliary labels to individual drug containers for each shipment were considered but determined to be infeasible for rapid deployment. In ACTIV-3, all study agents required dilution or reconstitution, so product as labeled was not seen by participants. For this reason, ACTIV-3 labeled all study products in English, the content of which would meet both US and European Union (EU) regulations [16,17]. For a country to participate in ACTIV-3, regulators had to accept this labeling as adequate for drug importation and use. This required collaboration between INSIGHT, its international coordinating centers (ICCs), and regulators. Label text translations were provided if needed by pharmacy staff. This decision to use single-language labels saved months of preparation for each agent while maintaining distribution flexibility where most needed. Translation of study documents related to IPs took significant time and cost to prepare; so, whenever possible a single language was used.

Documentation requirements for importing IPs and other required materials vary widely. Import and regulatory approval requirements for IPs and other materials from different countries should be evaluated in order to streamline regulatory processes. A centralized resource detailing country-specific requirements could encourage standardization and avoid regulatory delays.

Engagement of multiple regulators and other oversight bodies early in protocol development to obtain buy-in for nonstandard endpoints and procedures and commit to rapid clinical trial evaluation is essential.

A functioning multinational regulatory consortium should be established before the next pandemic to establish standards for national ethics and regulatory bodies for pandemic operations, encourage flexibility with requirements, expedite problem-solving, and facilitate regulator and ethics committee collaborations. The African Vaccine Regulatory Forum (AVAREF) [18] and the full implementation of the EU Clinical Trial Regulation via the Clinical Trial Information System (CTIS) [19] in 2023 forecast the possibility of such collaboration and procedure unification. Compilation of existing regulations on clinical trial operations would help with standardization. NIAID’s ClinRegs website [20] is an excellent resource for this information, but many countries involved in ACTIV efforts are not yet included.

## Investigational products (IPs) and supply

Proper IP management is highly regulated. For all ACTIV protocols, IPs were required to adhere to these regulations. These laws are the backbone for investigational drug service (IDS) pharmacies; however, many sites not affiliated with an academic medical center do not have dedicated research pharmacy services. Unfamiliarity and lack of site capacity (i.e., infrastructure, equipment, staffing), created many challenges for site pharmacies. Involving sponsor and network research pharmacists at protocol conception and prior to implementation as well as use of an assessment checklist for site pharmacies as discussed below will assist in identifying and mitigating such challenges.

Over the course of one master protocol, IPs could be added or subtracted at any time. Each IP required individual receipt, storage, dispensing, and documentation. While most US pharmacies had storage capacity for ambient and refrigerated (2°C–8°C) storage, some IPs required -20°C storage, which was not always readily available at non-US pharmacies. Proper temperature monitoring of each cold-chain unit was essential, and many sites required additional temperature data loggers to conform to manufacturer requirements. As soon as IPs are selected for a protocol, discussions between IP manufacturers and network research pharmacists about standard operating procedures (SOPs), pharmacy manuals, temperature excursion requirements, and other requirements will reduce potential issues during protocol initiation and study product administration.

Another challenge included proper sterile compounding equipment required for preparation of intravenous admixtures. In the US, this is a highly regulated area, including specialized air handling operations and infrastructure, proper engineering controls, and aseptic compounding technique; however, this presented a challenge for some sites, including those not catering to an infusion-needing population or sites without this costly equipment infrastructure.

IP-specific concerns were raised in ACTIV-3B. This IP had drug-specific issues, including a strict dosing scheme, tight control over administration, concerns about IP agglomeration, product compatibility, and manufacturing company switches. Dose was determined by narrow weight-based ranges (pmol/kg) requiring dilution of the IP and very low administration volumes. Due to agglomeration concerns, strict instructions were provided for filtration of the final solution and sourcing of compatible products (infusion bag, syringes, and infusion tubing). IP in-use period was very short, under 2 months, making it extremely challenging to distribute globally from a single US manufacturer. Finally, the IP manufacturer and manufacturing procedures were changed during the study, leading to different preparation SOPs, and creating additional burden. IP requirements of this nature should be avoided if possible under pandemic conditions.

For ACTIV-3, the DCR ICC created a checklist for pharmacy sites to assess capacity that could be a good model for future trials (Supplemental Material 1). This helped quickly identify sites with proper capacity to store compound sterilely and dispense IP with specific needs, which improved efficiency and availability to participate in testing each IP. In addition, sponsor support was available to sites lacking properly trained pharmacists, equipment, cold-chain monitoring, and training.

Regular pharmacist meetings across sites and with sponsor research pharmacists could decrease efforts spent by the study team while allowing subject matter experts to identify concerns, discuss, and formulate a strategy on how to implement difficult procedures. Many nuances regarding proper drug storage, temperature monitoring, preparation, and dispensing exist for which only IDS pharmacists have knowledge. In addition, most site pharmacists do not participate in larger network investigator and operations calls, therefore questions may not arise until the first participant is enrolled, delaying treatment. Often, minor concerns can be addressed or strategies shared among professional colleagues when dedicated time is allocated. [Further information on supply issues can be found in the ACTIV Inpatient Site-Specific Challenges manuscript.]

## Change of standard of care during trial

ACTIV-1, -2, -3, -4A/B/C trials started in August 2020 and expanded to ACTIV-3B, -4HT, -5, and -6 in 2021. During all ACTIV trials, protocol leadership aimed to maintain standard of care (SOC) during trials based on NIH COVID-19 treatment guidelines [21]. This was a big challenge as SOC evolved during the pandemic, causing protocols to be amended several times. Maintaining an adaptive platform with flexibility to adjust trial design took effort in many aspects (i.e. training staff, cost, recruitment, supply, IRB approval). Adaptive designs, discussed further in the ACTIV Statistical Lessons Learned manuscript, were justified by unpredictable changes requiring SOC adaptation and treatment guideline evolution.

## Centralized continuous investigator support

Complex protocols benefit from a pool of on-call investigators to troubleshoot questions with local sites; this model was more efficient than the traditional model of a single medical monitor from the sponsor. Call centers with twenty-four-hour service with designated sponsor on-call study team members or willing on-call investigatory staff were critical for information dissemination and key protocol questions in the majority of the ACTIV studies. In addition to outpatient and remote study enrollment, this will allow enrollment of inpatients and ICU trials to happen outside of normal working hours in multiple time zones. Weekly inclusive study team calls allowed for sharing of information, challenges, design needs, and questions about concurrent clinical care. These should be best practices for all large or complex master protocols in the future.

## Site training and virtual communications

Study initiation visits were conducted remotely and offered protocol implementation training, with in-depth training offered for more complex trial stages and to sites with risk-based identified training needs. Remote training session scheduling was challenging due to staff availability, time zone differences, and variety and volume in training needs. Recorded training sessions offered flexibility for site staff training needs. Meeting platforms (e.g., Skype, MS Teams, Zoom) posed challenges with site systems. ACTIV would recommend asynchronous training for future efforts. Several trials implemented weekly trouble-shooting calls for enrolling sites, with investigators and staff presenting their enrollment experience. Tools for communicating with study participants, such as flipbooks, which are pictorial documents used to describe the trial to potential participants during the informed consent process, proved to be valuable resources. Though some sites may still require hard-copy flipbooks for potential participants, moving flipbooks to an electronic platform improved utilization for some populations and should be considered for use in future trials, especially if those platforms are broadly accessible. [Further information on protocol implementation materials can be found in the article on practical application of good participatory practices and supplementary material in this supplement.] Meeting call options, such as toll-free phone calls, proved useful in ensuring access to protocol team meetings.

## Role of DSMB in trial implementation

During a health emergency, the independent Data and Safety Monitoring Board (DSMB) role takes on even greater importance, as they need to review rapidly evolving data and evaluate emerging safety, efficacy, and futility outcomes. The role of the DSMB in early futility assessment ensured effective sequential assessment of tested agents. It was helpful that ACTIV-2 and ACTIV-3 shared a DSMB since several investigational agents were used in both trials. For innovative phase 2/3 study designs and IPs with minimal preexisting safety information, enrollment was initially restricted, and early review of safety data by the DSMB was critical. Traditional Adverse Event (AE) schemas employed for registration trials are poorly adapted to a critically ill population and risk burying signal in noise; for AEs that are complicated to report and interpret (e.g., hypotension), a formal schema developed before trial launch is important. Some regulators were skeptical about whether the DSMB could adequately evaluate benefits and risks of an IP during trial conduct.

In ACTIV-3, the trial team placed all responsibility for evaluating efficacy and safety of IPs during trial conduct with the DSMB. This experienced group of external experts had access to unblinded trial data and external information and was able to compare outcomes between participants receiving active medication and those receiving placebo. The DSMB was also knowledgeable about background clinical complication rates from underlying conditions, and hence able to evaluate whether accumulation of such conditions within a trial reflected the type of participants enrolled (e.g., the underlying disease rate) versus an emerging safety signal from the IP. While ACTIV-3 was blinded, only the DSMB was unblinded to treatment assignments: evaluation and reporting of Suspected Unexpected Serious Adverse Reactions by sites and medical monitors was done blinded, and the ACTIV-3 sponsor received only reported Serious Adverse Events, Unanticipated Problems, and Adverse Event of Special Interest.

There was an instance in one ACTIV-3 trial where the DSMB advised continuing enrollment to the IP, but the FDA requested the trial stop enrolling. Despite efforts to enable the DSMB to discuss with the FDA, the trial was halted. Retrospectively it was not clear the trial should have been stopped. Additional policies for health emergencies and dialog between regulators and DSMBs are warranted.

## Risk management plan for continuity of support during trial

When external resources are provided, risk management and transition plans are needed to ensure continuity through program completion, and there is no lapse in needed network support. For example, CombatCOVID, the only cross-USG trial website and communications tool for recruitment to the ACTIV and other USG COVID-19 trials, was created by the HHS in December 2020. It provided a central website for information about the trials and allowed for rapid on-site and remote site staffing and resource augmentation. This support was terminated with short notice with no transition plan, leaving the burden on the individual trials to develop and maintain separate websites and other recruitment tools after December 2021.

## Participant follow up

Drawing blood from participants who had been discharged proved challenging for inpatient trials. Concerns existed about contagiousness; in some institutions, participants were not allowed to enter clinics to have blood and other samples collected. Many participants also found it difficult or ill-advised to travel to clinics for follow-up visits. Providing a phlebotomy vendor or site staff for home blood draws was helpful in managing these challenges, making the trial more participant-friendly, and should be implemented early in trials.

Inpatient sites needed free beds and subsequently transitioned participants to long-term care facilities during hospitalizations, which posed challenges with determining endpoints related to hospitalization status. Future trials should include follow-up location options, including long-term care, outpatient pharmacy clinics [22], mobile research vans, and other creative options devised to ensure data collection for the study duration.

Severely ill hospitalized patients have many AEs and abnormalities in laboratory tests during their illness. This greatly increases AEs reporting burden. Protocol teams and regulatory agencies should consider which AEs, and at what grades, are necessary to report.

## Conclusion

The ACTIV program was a unique exercise with many lessons to help during future clinical trial implementation. Here, we propose an established, well-coordinated, and functioning multinational network infrastructure for governments and international organizations with the ability to implement prioritized large-scale clinical trials to be in place and prepared for future global health emergencies. It is also critical for governments, regulatory authorities, and other non-network stakeholders to address the challenges to study implementation identified, harmonize their processes, remove or reduce barriers and delays, and ensure that, in future health emergencies, trials to identify beneficial medical countermeasures can be developed and implemented as expeditiously and effectively as possible. Lessons learned and recommendations for pandemic master protocol implementation are summarized in Figure 2.

## Supporting information

Keshtkar-Jahromi et al. supplementary material Keshtkar-Jahromi et al. supplementary material

## Supplementary material

The supplementary material for this article can be found at https://doi.org/10.1017/cts.2024.507.
