# Supplementary material for: ACTIV trials: cross-trial lessons learned for master protocol implementation
Source: J Clin Transl Sci. 2024 Oct 15;8(1):e152. doi: 10.1017/cts.2024.507 (PMC11557279; doi:10.1017/cts.2024.507)
Supplement: Keshtkar-Jahromi et al. supplementary material [file S2059866124005077sup001.pdf]

# PHARMACY VISIT CHECKLIST

| | |
|---|---|
| **PROTOCOL TITLE**: ***A MULTICENTER, ADAPTIVE, RANDOMIZED, BLINDED CONTROLLED TRIAL OF THE SAFETY AND EFFICACY OF INVESTIGATIONAL THERAPEUTICS FOR HOSPITALIZED PATIENTS WITH COVID-19*** | |
| **PROTOCOL NUMBER:** INSIGHT14/ TICO | **SITE PRINCIPAL INVESTIGATOR'S NAME:** |
| **SITE NAME/NUMBER:** | **LEAD PHARMACIST NAME:** |
| **VISIT CONDUCTED BY:**<br><br>**DATE OF (REMOTE) VISIT:** | **METHOD OF VISIT: ZOOM ☐ FACETIME☐ OTHER☐:-**<br>__________________ |
| **LIST STUDY PRODUCTS AT TOUR:** | **PHARMACY TOUR NUMBER:** ___________ |

**General Visit Overview:**

- Review: introductions, discuss general information, and any initial questions for pharmacists.
- Determine access to the INSIGHT website, passwords, explain distribution of the pharmacy login, area of the website where the pharmacy study materials are posted along with study drug procedures.
- Ask about protocol training attendance for all pharmacists and ability to conduct training for future pharmacists.
- Ask about pharmacy SOPs (names) and study SOPs (any written ones they have that are study specific-make a note of it).
- Discuss the importance of pharmacy procedures, keeping the blind, treatment assignment, electronic prescription and requirement for signed paper prescriptions form with a wet signature.
- Discuss pharmacy monitoring and accountability logs.
- Confirm receipt of study product and acknowledgment, as applicable.
- Ask the pharmacists to walk around with the mobile device and show the facilities. Ensure this includes a review of security, alarm system, ambient temperature storage units, refrigerators, and emergency power.
- Remind pharmacists that all questions are to be addressed to the DCR ICC and that the ultimate responsibility for the pharmacy conduct is the Principal Investigator.

# PHARMACY VISIT CHECKLIST

*Instructions: Complete the yes/no/ boxes if applicable to the question and if not mark NA; complete notes/comments field as applicable.*

| | Study procedures to be discussed and verified during the initiation visit (by the Sponsor or Designee) | Yes | No | NA | Notes/Comments |
|---|---|---|---|---|---|
| | 1. Where is the pharmacy located, within the inpatient or outpatient pharmacy? Does the site rely on an external research pharmacy? | | | | |
| | 2. Is the address the same as the site address? How far is the pharmacy from the inpatient unit? | | | | |
| | 3. Is there a temperature recording device for storage location? What method of temperature recording is utilized? | | | | |
| | 4. Is there electronic temperature monitoring for refrigerators (at a minimum every 60 minutes), electronic monitoring for ambient temperature, and any other medication storage areas such as freezers or medicine cabinets where investigational products or placebo may be stored? Who is notified if there is a temperature excursion? How is that person notified? | | | | |
| | 5. Does the site have designated freezers for future study agents? If yes how are they monitored? | | | | |
| | 6. Area locked and secured with limited access? Who has access to IP? | | | | |
| | 7. Will medications be segregated from other hospital meds? How will the inventory be kept? | | | | |
| | 8. Where will the infusion be prepared: Sterile room? Clean room? Biosafety cabinet? Fume hood? | | | | |
| | 9. What is the availability of emergency power (e.g. generator)? Are all applicable storage devices on back-up power? If not, explain. | | | | |
| | 10. Is there a back-up refrigerator/freezer/ secure location for study medication? If so, do they have temperature monitoring, alarms and back-up power? | | | | |

# PHARMACY VISIT CHECKLIST

| Study procedures to be discussed and verified during the initiation visit (by the Sponsor or Designee) | | Yes | No | NA | Notes/Comments |
|---|---|---|---|---|---|
| | 11. How often is the equipment used for the study such as flow hoods and cold-chain equipment serviced and calibrated? Is the service and calibration current? | | | | |
| | 12. Special security procedures reviewed (e.g. for investigational products)? | | | | |
| | 13. Are equipment and facilities adequate as required by the protocol? | | | | |
| | 14. Did the pharmacy receive the amber bags for blinding study IP? | | | | |

# PHARMACY VISIT CHECKLIST

| | | | | | |
|---|---|---|---|---|---|
| **Pharmacy Staff** | | | | | |
| | 1. Document the names of the pharmacy staff in attendance. Are there any additional staff that need to be added to the pharmacy plan? | | | | |
| | 2. Did the back-up pharmacists take the protocol training? Will the site require availability of the pharmacists for weekend enrollments? Encourage pharmacists to discuss with PI. | | | | |
| | 3. Who has access to the pharmacy area and facilities? What security features are in place for the facilities? | | | | |
| | 4. Who will prepare the infusion? How are technicians supervised? | | | | |
| | 5. Who delivers the infusion? Does the pharmacy use a chain of custody form? | | | | |
| | 6. Has the pharmacy encountered problems ordering supplies? Does the pharmacy need anything in particular? | | | | |
| **Pharmacy files and documentation** | | | | | |
| | 1. Does the pharmacy have a copy of the current pharmacy plan? Any changes in pharmacy plan are to be report to the DCR ICC. | | | | |
| | 2. Does the pharmacy have the protocol training slides and PIM available for all? | | | | |
| | 3. Will the monitors have access to the pharmacy facilities? Will monitoring be done via virtual visit? What is the hospital allowing during COVID? | | | | |
| | 4. What is the availability of source data and pertinent documentation for the protocol requirements? | | | | |
| | 5. How are accountability records and acknowledgment of receipts maintained? | | | | |
| | 6. Was the flow of paperwork through facility from request of study agent through study agent destruction reviewed? | | | | |

# PHARMACY VISIT CHECKLIST

**Additional General comments: (number items if needed and delete extra rows if not needed)**

| |
|---|
| |

______________________________________________

**Signature of Author/Date (Leader of tour/call)**

______________________________________________

**Signature of Designee/ Date**

**(If RCHSPP/CTM lead tour, CTM Manager designee to sign; if not fill in NA)**

**Note:**

*Electronic signatures using a PIV card is acceptable*
